# Contribution of engineered nanomaterials physicochemical properties to mast cell degranulation

**DOI:** 10.1038/srep43570

**Published:** 2017-03-06

**Authors:** Monica M. Johnson, Ryan Mendoza, Achyut J. Raghavendra, Ramakrishna Podila, Jared M. Brown

**Affiliations:** 1 Department of Pharmaceutical Sciences, Skaggs School of Pharmacy and Pharmaceutical Sciences, University of Colorado, Aurora, CO 80045, USA; 2 Department of Physics and Astronomy, Clemson University, Clemson, SC 29634, USA; 3 Clemson Nanomaterials Center and COMSET, Clemson University, Anderson, SC 296225, USA

## Abstract

The rapid development of engineered nanomaterials (ENMs) has grown dramatically in the last decade, with increased use in consumer products, industrial materials, and nanomedicines. However, due to increased manufacturing, there is concern that human and environmental exposures may lead to adverse immune outcomes. Mast cells, central to the innate immune response, are one of the earliest sensors of environmental insult and have been shown to play a role in ENM-mediated immune responses. Our laboratory previously determined that mast cells are activated via a non-FcεRI mediated response following silver nanoparticle (Ag NP) exposure, which was dependent upon key physicochemical properties. Using bone marrow-derived mast cells (BMMCs), we tested the hypothesis that ENM physicochemical properties influence mast cell degranulation. Exposure to 13 physicochemically distinct ENMs caused a range of mast degranulation responses, with smaller sized Ag NPs (5 nm and 20 nm) causing the most dramatic response. Mast cell responses were dependent on ENMs physicochemical properties such as size, apparent surface area, and zeta potential. Surprisingly, minimal ENM cellular association by mast cells was not correlated with mast cell degranulation. This study suggests that a subset of ENMs may elicit an allergic response and contribute to the exacerbation of allergic diseases.

Nanotechnology has grown exponentially over the last several decades, spurring the rapid development of engineered nanomaterials (ENMs) for applications in markets including technology, consumer products, and nanomedicines. The unique properties are useful for improving product formulations and efficacy in targeted imaging and drug delivery^1^,^2^. However, due to the increased exposure from extensive manufacturing and incorporation into consumer products, concerns are raised regarding ENM safety and effect on human and environmental health^3^.

The field of nanotoxicology has begun addressing these concerns and it has become clear that the physicochemical properties of ENMs (size, chemical composition and stability, morphology, surface modifications, etc.) contribute to both desired and undesired biological outcomes^4^,^5^. Our increased understanding of the role for physicochemical properties in biological responses to ENMs will improve safety, however it presents a grand challenge for the field as the number of ENMs and physicochemical modifications continue to rapidly expand. ENMs can be manipulated and manufactured with different sizes, shapes, surface modifications, structural and chemical defects. Several studies observed the dependency of ENM size and surface coating on cellular uptake and membrane internalization^6^,^7^. For example, Mukherjee *et al*., demonstrated that the ENM-ENM interactions as well as the ENM interaction with the biological environment influence ENM agglomeration, dissolution, diffusion, and sedimentation^8^. These changes are inevitably affecting ENM biodistribution and toxicity in biological systems^9^,^10^. Once ENMs enter a biological environment, they are enveloped by proteins, lipids, and macromolecules forming a biocorona, which could drastically modify ENM-bio interactions^11^,^12^. The formation of a biocorona adds an additional layer of complexity in assessing toxicity due to modification of surface properties of the ENMs. More research is needed to correlate ENM properties to the physiological responses after exposure but this has become exceedingly difficult due to the lack of standardized dosimetry methods and characterization reported in cytotoxicity studies.

More effort needs to be focused on allergic responses to ENMs, as this remains a likely immune outcome particularly for metal-based nanoparticles^13^,^14^,^15^. Studying adverse immune responses following ENM exposure has become increasingly important with emergence of nanomedicines, considering drug allergies are a limiting factor for patient compliance and could often lead to drug removal from the market^16^. Mast cells, involved in the innate and adaptive immune responses functioning in host defense, have been implicated as an early effector cell in response to ENM exposures^17^,^18^,^19^,^20^,^21^,^22^,^23^,^24^,^25^,^26^,^27^. They play a predominant role in the development of allergic inflammation, resulting in diseases such as allergies, asthma, and anaphylaxis. In addition, mast cells are involved in wound healing, fibrosis, cardiovascular disease, and autoimmunity^17^,^18^,^28^. Mast cells are activated via crosslinking of high-affinity immunoglobulin E (IgE) receptors (e.g. FcεRI) upon challenge with an allergen. In contrast, they can also be activated via non-IgE- ligands (e.g. toxins, complement components) through various surface receptors (e.g. toll-like receptors, complement receptors, scavenger receptors), or potentially unknown mechanisms^29^,^30^,^31^,^32^,^33^,^34^. Activation corresponds to the release of preformed granules (degranulation) and *de novo* synthesized mediators (activation) such as histamine, serotonin, proteases, cytokines (TNF-α, TGF-β, IFN-γ, IL-1β, IL-4, IL-9, IL-13, IL-33), and osteopontin (OPN).

Recent studies have demonstrated the role of mast cell infiltration and activation in response to ENM exposures. Studies have shown that mast cells contribute to ENM-mediated lung inflammation and adverse cardiovascular health effects^23^,^35^. In addition, mast cell-deficient mice were protected from pulmonary inflammation following cerium oxide nanoparticle instillation^36^. Wang *et al*. demonstrated that TiO_2_ nanoparticles induced mast cell recruitment in the gastrointestinal tract following a 30 d exposure in Sprague-Dawley rats^37^. Similarly, Chen *et al*., observed an increase in histamine release and serum IgE upon TiO_2_ exposure *in vitro*^24^. Oxidative stress was shown to play a role in mast cell-mediated dermal toxicity by single-walled carbon nanotube exposure^19^. In addition, some ENMs have been shown to be negative regulators of mediator release in mast cell degranulation. For example, fullerenes were shown to inhibit IgE-mediated^38^ and non-FcεRI-mediated mast cell degranulation and activation, which was dependent on the presence of key moieties and side-chains^39^. We recently reported that mast cell degranulation occurred after an acute exposure to silver nanoparticles (Ag NPs), and was dependent on key physicochemical properties such as size and surface coating^40^. These and several other studies outlined the ability of ENMs to activate and inhibit mast cell responses in a variety of exposure models. Notwithstanding these results, the underlying mechanisms of ENM-mast cell interactions and the key ENM physicochemical properties in mast cell activation remain unknown.

In this study, we aimed to characterize the physicochemical properties of a library of ENMs and measure mast cell degranulation and activation following exposure to identify properties that may potentially exacerbate an allergic response. We have demonstrated that different ENMs cause a wide range of mast cell responses. ENM physicochemical properties such as size and stability that contribute to total particle surface area were identified as contributing factors in mast cell degranulation.

## Results

### ENM characterization

The hydrodynamic size and zeta potential of ENMs were measured in both diH_2_O & HEPES buffer, the biological buffer used for subsequent *in vitro* assays (Table 1). The shape and size of all ENMs were further confirmed by TEM images (Fig. S1). As expected, the hydrodynamic size in both diH_2_O and HEPES was significantly higher than the dehydrated size measured using TEM for all ENMs. The largest size difference was observed with TiO_2_, the primary size measuring 49 nm and the hydrodynamic size measuring 696 nm (diH_2_O) and 979 nm (HEPES) suggesting the presence of TiO_2_ aggregates. In addition, we observed significant agglomeration for MgO, SiO_2_-30 and SiO_2_-60 based on their large hydrodynamic size relative to the dehydrated TEM size. All ENMs except the four Ag NPs evaluated had low zeta potentials indicating reduced suspension stability. Using the hydrodynamic sizes of each ENM, apparent surface area and total particle number (per gram) were calculated in both diH_2_O and HEPES (Table 1). Ag-5 and Ag-20 had the largest apparent surface areas and particle numbers per gram in diH_2_O. The next largest surface area was observed with CuO, measuring at 10.96 m^2^/g. However, the surface area was drastically decreased once the particles were diluted in HEPES buffer due to agglomeration.

### ENMs induce minimal cytotoxicity in bone marrow-derived mast cells

**Figure 1:**
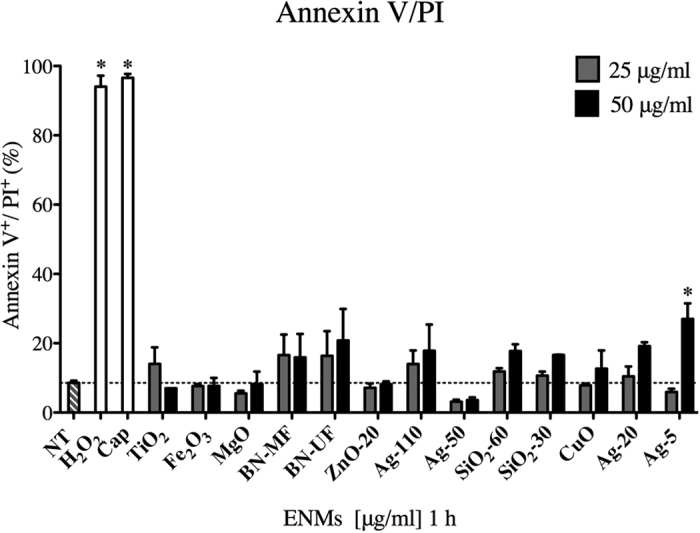
Cytotoxicity of ENMs. Bone-marrow-derived mast cells (BMMCs) exposed for 1 h to ENMs at 25 & 50 μg/ml were analyzed via flow cytometry for Annexin V/propidium iodide (PI) expression on the surface of apoptotic cells. Hydrogen peroxide (H_2_O_2_, 10 mM) and capsaicin (Cap, 100 μM) were used as positive controls (white bars). Horizontal dash line represents NT control values. Values are expressed as mean ± SEM (N = 3/group). *Indicates significant difference between non-treated control group (*p* ≤ 0.05).

Cytotoxicity of BMMCs following exposure to ENMs was evaluated by flow cytometric analysis measuring Annexin V and propidium iodide (PI; Fig. 1). Importantly, BMMCs treated with 25 and 50 μg/ml ENMs for 1 h (time point at which all degranulation studies were performed) showed minimal number of cells undergoing apoptosis or necrosis as measured by Annexin V and PI respectively, further confirming that ENMs are not activating mast cells through a cytotoxic mechanism.

### ENM cell association by mast cells

**Figure 2:**
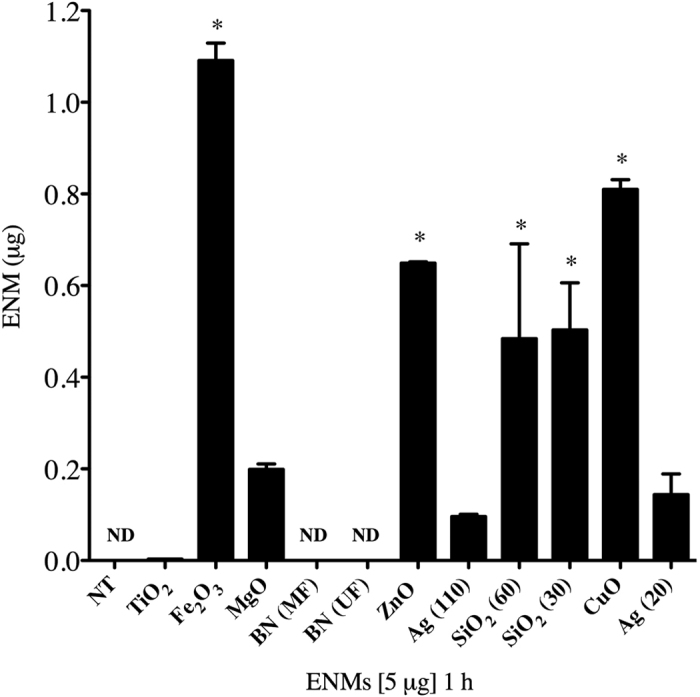
ICP-MS measurement of cell association of ENMs by bone-marrow-derived mast cells (BMMCs). Mast cell association of ENMs was evaluated following 1 h exposure of 5 μg of ENMs. Values are expressed as mean ± SEM (n = 3/group). *Indicates significant difference between non-treated control groups (*p* ≤ 0.05). ND indicates non-detectable.

We next set out to assess the role of ENM cellular association by mast cells and its contribution to mast cell degranulation (Fig. 2). Following ENM exposure, we observed significant cell association of Fe_2_O_3_, ZnO, SiO_2_-30, SiO_2_-60, and CuO. BN-MF and BN-UF cell association was below the limit of detection. Interestingly, Ag-20 and Ag-110 were not readily related to mast cell association as compared to the other particles analyzed, suggesting that cell association may not be contributing to mast cell degranulation.

### ENMs induce mast cell degranulation

**Figure 3:**
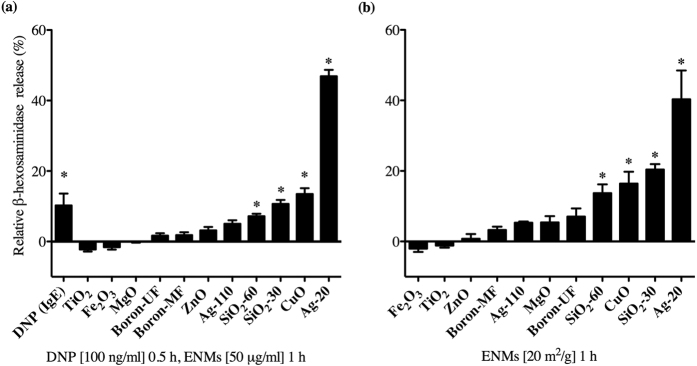
ENM-mediated mast cell degranulation. Bone marrow-derived mast cells (BMMCs) were exposed to ENMs for 1 h and evaluated for the release of β-hexosaminidase into the supernatant. (**a**) BMMCs were exposed to (**a**) mass concentrations of ENMs (50 μg/ml) or DNP (100 ng/ml) as an IgE positive control and (**c**) ENMs normalized to surface area (20 m^2^/g). Values are expressed as mean ± SEM normalized to non-treated control group (n = 3/group). *Indicates significant difference from non-treated control group (*p* ≤ 0.05).

**Figure 4:**
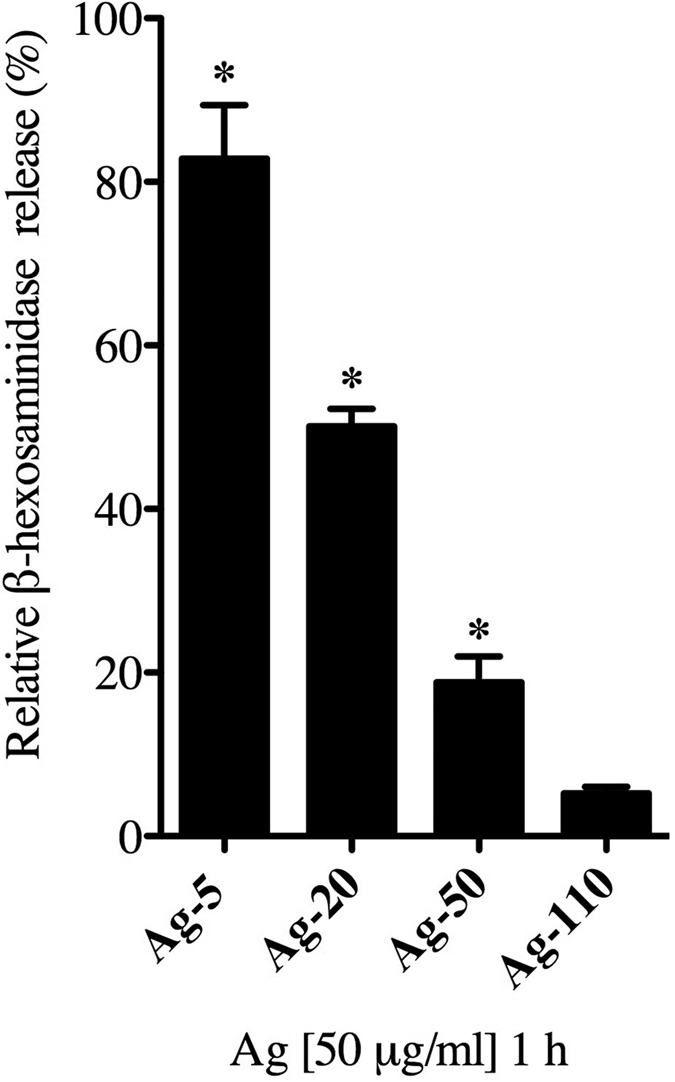
Size dependency of Ag NP-mediated mast cell degranulation. Bone marrow-derived mast cells (BMMCs) were exposed to 50 ug/ml of Ag NPs at different sizes (5, 20, 50, 100 nm) for 1 h and evaluated for the release of β-hexosaminidase into the supernatant. Values are expressed as mean ± SEM normalized to non-treated control group (n = 3/group). *Indicates significant difference from non-treated control group (*p* ≤ 0.05).

Mast cell degranulation 1 h post exposure to 50 μg/ml of ENMs was assessed by measuring the release of β-hexosaminidase in the supernatant (Fig. 3). In addition, FcεRI crosslinking by dinitrophenyl (DNP) was assessed as a positive control. Ag-20 caused the most dramatic degranulation of mast cells (47%) as measured by β-hexosaminidase release, followed by CuO (14%), SiO_2_-30 (11%), and SiO_2_-60 (7%), respectively (Fig. 3a). These particles demonstrated a dose-dependent increase in degranulation at the doses 25, 50, and 100 μg/ml (Fig. S2). Interestingly, TiO_2_ and Fe_2_O_3_ caused an inhibition of mast cell degranulation, however this was not statistically significant. MgO, BN-MF, BN-UF, ZnO, and Ag-110 were not found to cause significant mast cell degranulation compared to non-treated control group (Fig. 3a). Furthermore, we demonstrated that smaller Ag nanoparticles induced the highest percent mast cell degranulation (5 nm, 82%), decreasing in response with an increase in particle size (5, 20, 50, 110 nm; Fig. 4).

In addition to exposing mast cells to a mass-based concentration of ENMs (μg/ml), we also took into account the changes in hydrodynamic size of each ENM and treated BMMCs with ENM doses normalized to the same total surface area. Apparent surface area was calculated for each ENM using particle densities and hydrodynamic size in HEPES buffer. To evaluate the role of surface area in mast cell response, BMMCs were treated for 1 h with ENMs normalized to total surface area of 20 m^2^/g (Fig. 3b). The results were similar to the mass-based concentration dosing, with Ag-20 causing the most abundant mast cell degranulation (40%), followed by SiO_2_-30 (20%), CuO (16%), and SiO_2_-60 (14%). All other ENMs did not cause significant degranulation compared to control. Overall, we observed a higher percent mast cell degranulation when dosing based on surface area, however the trend did not change when compared to mass based concentration dosing at 50 μg/ml.

### Formation of an ENM-biocorona reduces mast cell degranulation

**Figure 5:**
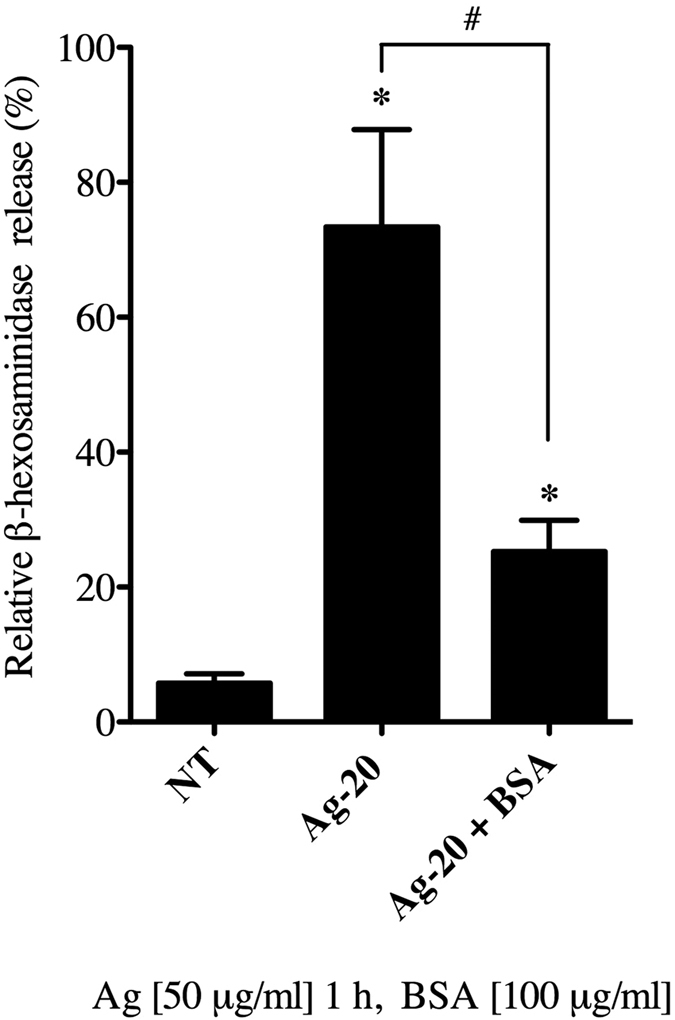
Influence of an ENM-biocorona on mast cell degranulation. Bone marrow-derived mast cells (BMMCs) were exposed to 20 nm-sized Ag NPs (Ag-20) with or without the addition of a bovine serum albumin (BSA) biocorona at a concentration of 50 μg/ml for 1 h and evaluated for the release of β-hexosaminidase into the supernatant. Values are expressed as mean ± SEM (n = 3/group). *Indicates significant difference from non-treated control group and ^#^indicates significant difference between groups with and without a biocorona (*p* ≤ 0.05).

To evaluate the influence of silver nanoparticle-induced mast cell degranulation in a biologically relevant condition, Ag-20 (20 nm) were coated with bovine serum albumin (BSA) to represent a normal serum component and mast cell degranulation was assessed. Formation of a biocorona on Ag-20 resulted in an increase in the hydrodynamic size and decrease in nanoparticle stability in suspension evident by a lower zeta potential as compared to Ag-20 without a biocorona (Table 2). Interestingly, the addition of a BSA biocorona on Ag-20 led to a significant reduction in mast cell degranulation compared to treatment of Ag-20 alone (Fig. 5).

### ENMs did not exacerbate IgE-mediated mast cell degranulation

**Figure 6:**
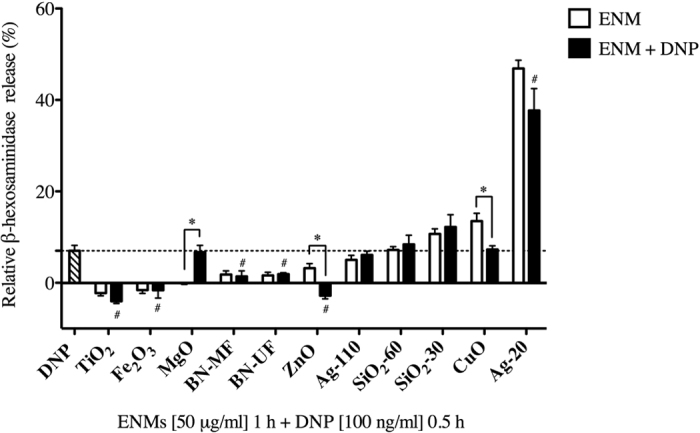
Impact of ENM exposure on IgE meditated mast cell degranulation. BMMCs were exposed to ENMs (50 μg/ml) for 1 h followed by addition of DNP treatment (100 ng/ml) (black bars) and evaluated for the release of β-hexosaminidase into the supernatant. Values are expressed as mean ± SEM normalized to non-treated control group (n = 3/group). *Indicates significant difference between ENM-treated (white bars) and ENM + DNP-treated groups (black bars) (*p* ≤ 0.001). ^#^Indicates significant difference between DNP-treated group (striped bar and dashed line) and ENM + DNP- treated groups (black bars) (*p* ≤ 0.05).

Next we aimed to evaluate if ENM exposure contributes to IgE-mediated mast cell degranulation in an allergic response. BMMCs were sensitized overnight with IgE anti-DNP, and subsequently treated with ENMs for 1 h (50 μg/ml) followed by stimulation of FcεRI by DNP (100 ng/ml) for 30 min to stimulate IgE- mediated mast cell degranulation (Fig. 6). All ENMs except MgO, ZnO, and CuO did not demonstrate a significant difference in mast cell degranulation when comparing ENM treatment alone to ENM treatment with DNP stimulus (IgE-mediated), suggesting that a subset of ENMs are not interfering with IgE-mediated activation. Surprisingly, CuO inhibited the IgE response when compared to ENM treatment alone. In addition, compared to DNP treatment alone, TiO_2_, Fe_2_O_3_, and ZnO all reduced IgE-mediated degranulation. Overall, mast cell degranulation varies depending on the ENM type, suggesting an essential role for physicochemical properties in mast cell response.

### ENM physicochemical properties associated with mast cell degranulation

**Figure 7:**
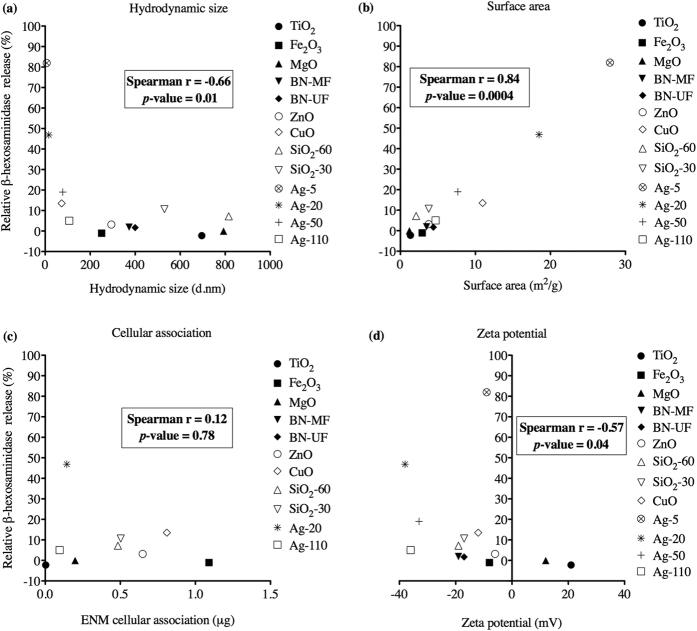
Statistical association calculated between mast cell degranulation and ENM size, apparent surface area, zeta potential, and cellular association. Correlation using Spearman’s rank-order test was performed on data from bone marrow-derived mast cells (BMMCs) treated with 50 μg/ml of ENMs plotted against (**a**) ENM hydrodynamic size (nm), (**b**) surface area (m^2^/g), (**c**) cell association (μg) and (**d**) zeta potential (mV) following exposure. Spearmans r and *p*-value are reported in insert. Differences were considered statistically significant at *p* ≤ 0.05.

Spearman’s rank-order test was performed to determine the strength of association between mast cell degranulation (data derived from Figs 3a and 4) and factors including size, stability, apparent surface area, and cell association following exposure (Fig. 7). ENM surface area showed a significant correlation with degranulation (Spearman r = 0.84, *p* = 0.04 × 10^−2^), in addition to hydrodynamic size (Spearman r = −0.66, *p* = 0.01), indicating that mast cell degranulation increased with smaller particle size (Fig. 7a and b, respectively). Zeta potential, also significant, had a negative correlation with mast cell degranulation (Spearman r = −0.57, *p* = 0.04). Contrary to expectations, cell association of ENMs did not have a significant correlation with mast cell degranulation (Spearman r = 0.12, *p* = 0.78).

### Late-phase mast cell activation following ENM exposure

**Figure 8:**
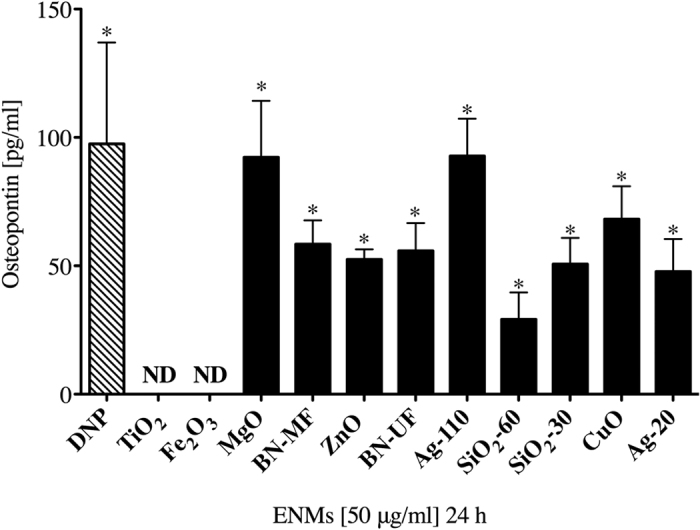
Osteopontin levels were measured in supernatants of BMMCs treated with ENMs by ELISA. BMMCs were treated with ENMs at 50 μg/ml or DNP at 100 ng/ml as IgE-mediated positive control (stripped bar) for 24 h. Values are expressed as mean ± SEM normalized to non-treated control group (n = 3/group). ND indicates not detected. *Indicates significant difference from non-treated controlled group normalized to 0 (*p* ≤ 0.05).

In addition to assessing immediate phase mast cell activation (i.e. degranulation) following ENM exposure, late-phase mast cell activation was evaluated by measuring the release of osteopontin (OPN), a *de novo* synthesized mediator which was previously shown to be released in response to Ag-20^40^. OPN was measured in the supernatant of BMMCs treated for 24 h with ENMs at 50 μg/ml or DNP at 100 ng/ml (n = 3/group) (Fig. 8). Interestingly, OPN was detected in supernatants of BMMCs exposed to DNP and all ENMs except TiO_2_ and Fe_2_O_3_, suggesting that early phase mast cell degranulation is not indicative of late-phase mast cell activation following ENM exposure.

## Discussion

Engineered nanomaterials (ENMs) with various physicochemical properties (size, chemical composition and stability, morphology, surface modifications, etc.) have been previously shown to contribute to pathophysiological responses, however, it remains to be elucidated how these properties play a role in mast cell activation in an allergic response^5^. Our previous studies demonstrated the role of mast cells in the contribution to inflammation and pathology induced by cerium oxide and carbon-based ENM exposures^23^,^36^. More recently, we have shown that mast cells are activated in response to silver nanoparticles (Ag NPs) in a size and shape dependent manner^40^. The current study investigated the direct interactions of 13 physicochemically distinct ENMs on mast cell activation and degranulation. In summary, we determined that ENMs could induce mast cell degranulation with varying degrees of response depending on the type of ENM and its physicochemical properties. Furthermore, this degranulation response was driven by ENM surface area and not a result of cell association. Our study has provided evidence suggesting a role of ENM physicochemical properties in the activation of adverse mast cell responses.

The library of ENMs utilized in this study were chosen due to their incorporation into pharmaceutical, industrial, and commercial products. ENMs were fully characterized for their size, shape, and stability. Measurement of hydrodynamic size in both de-ionized water (diH_2_O) and a biological buffer (HEPES) demonstrated that select ENMs were likely agglomerating in solution, leading to changes in apparent surface area and total particle number. Changes in particle size and shape due to agglomeration have been shown to significantly alter ENM reactivity and toxicity^41^. Agglomeration can be the result of ENM-ENM interactions due to surface collisions, chemical transformations (i.e. oxidation/reduction, dissolution, degradation) or potential interactions with substances in the biological systems (i.e. proteins, macromolecules)^42^. Our current study demonstrated that an increase in particle size resulted in a decreased mast cell response. This notion was further confirmed in reduced mast cell degranulation observed following treatment with Ag-20 coated with a bovine serum albumin (BSA) biocorona in which nanoparticle size was increased as compared to Ag-20 without a biocorona. Overall, this suggests that stable ENMs not forming large aggregates are likely mediating the observed response.

Mast cells were utilized as the effector cell type specifically for their role in allergic disorders such as asthma, allergic rhinitis, atopic dermatitis and anaphylaxis^18^. Located near the host-environment interface, mast cells represent one of the first cell types to likely encounter ENMs^17^. Aside from occupational or industrial exposures, the emergence of nanomedicines has added a layer of difficulty in evaluating potential adverse immune interactions to ENMs. ENMs are currently being formulated and tested as drug delivery carriers due to their high surface area and ability to bind various compounds and ligands^43^. Knowledge about their potential toxicity and health impact is essential before these nanomaterials can be used in a clinical setting, as an allergic immune response is often a limiting factor in patient compliance. For example, a clinical trial evaluating the safety and efficacy of Ferraheme, an iron oxide nanoparticle-coated drug used for the treatment of iron anemia in chronic kidney disease patients, demonstrated hypersensitivity reactions as a major safety concern^44^. To date, there are limited toxicological screening methods to evaluate immune cell activation, specifically an allergic immune response, following ENM exposures or administration of nanomedicines.

In the current study, 20 nm Ag nanoparticles (Ag-20) had the highest stability measured by zeta potential, largest apparent surface area, and subsequently caused the highest degranulation of mast cells. In addition, the robust response to Ag NPs was significantly higher than all other ENMs evaluated as well as being higher than our IgE-mediated (DNP) positive control. Furthermore, mast cell response decreased as particle size increased with no observable response following exposure to 110 nm Ag, further confirming the role of ENM surface area in inducing a mast cell response. Ag NPs are one of the most utilized ENMs due to their antimicrobial properties and are incorporated into over 300 commercially available consumer products^45^. ENMs utilized as antimicrobial agents often rely on ionic dissolution of select metal-based particles such Ag, zinc, and copper nanoparticles, however ion dissolution has also been linked to toxicity^14^,^46^,^47^. For example, Wang *et al*., demonstrated a role for smaller Ag NPs (20 nm) to induce cytotoxicity due to a higher rate of dissolution compared to larger Ag NPs (110 nm) *in vivo*^9^. Although our current study did not directly measure ion dissolution resulting in mast cell degranulation, our laboratory previously reported that Ag^+^ ions alone did not induce mast cell degranulation, leading us to believe that ion dissolution is not playing a significant role in ENM-mediated mast cell responses^40^.

Canonical activation of mast cells via FcεRI crosslinking is primarily through an IgE-dependent mechanism, however our results suggest that select ENMs are activating mast cells through a non-IgE, potentially novel mechanism. Toll-like receptors, complement receptors, and scavenger receptors have been implicated in non-IgE mediated mast cell degranulation following exposure to unknown ligands^30^,^32^,^33^,^40^. Huang *et al*., has demonstrated a role for gold nanoparticles to bind and aggregate IgE receptors in the absence of IgE^20^. Our laboratory, as well as others has demonstrated the role for scavenger receptors in ENM-mediated mast cell activation *in vivo* and *in vitro*. Brown *et al*., identified a scavenger receptor involved in mast cell activation by crystalline silica in an SR-A/MARCO knockout mouse model^32^. Recently, we have shown that the scavenger receptor B1 is involved in Ag NP (20 nm)-induced mast cell degranulation and could be prevented using a tyrosine kinase inhibitor^40^. Our current study provides evidence that ENM-induced mast cell degranulation is not through an IgE-mediated response. In addition we speculate that due to lack of internalization, demonstrated in this study as well as other studies performed in our laboratory^40^,^48^, Ag NPs are likely modulating surface receptors or non-specific cell membrane interactions leading to the downstream activation of signal transduction pathways.

We demonstrated that exposure to the ENMs evaluated in the current study did not lead to the exacerbation of an allergic response evaluated by IgE-induced mast cell degranulation. However, TiO_2_, Fe_2_O_3_, and ZnO nanoparticles were shown to inhibit IgE-mediated mast cell degranulation to an allergen (DNP). Feltis *et al*., also demonstrated that ZnO inhibited IgE-dependent mast cell degranulation in a size-dependent matter^49^. ZnO was also shown to cause down-regulation of pro-inflammatory markers as well as inhibition of anti-DNP IgE-induced anaphylaxis in a mouse model^50^. Carbon-based ENMs (C_60_), which alone did not cause a response, were also shown to inhibit IgE-dependent mast cell activation through the reduced activation of global signaling molecules leading to decreased oxidative stress^38^. Our findings as well as others provide evidence that TiO_2_, Fe_2_O_3_, and ZnO nanoparticles could inhibit an allergic response mediated by mast cell activation.

The current study demonstrates that TiO_2_ is causing inhibition of mast cell degranulation upon exposure however this is in direct opposition to other studies showing that TiO_2_ plays a role in immunomodulatory activities^51^. Wang *et al*., demonstrated an increase in mast cell infiltration in response to TiO_2_ exposure *in vivo*, however showed no observable increase in histamine release, indicating that mast cells were recruited but not activated^37^. In addition, TiO_2_ exposure caused a dose-dependent increase in histamine release in a rat mast cell line (RBL-2H3), however the doses (0.1–1000 mg/ml) were much higher than the current study’s experimental doses (<100 μg/ml). This study also reported a much smaller hydrodynamic size for TiO_2_ at 83 nm and larger surface area of 46.3 m^2^/g, which could account for the difference in mast cell degranulation compared to our results^24^. The variation in results of our study compared to other studies could be accounted for by the difference in dosimetry and surface modifications, as other studies used much higher doses (>100 μg/ml) and were also coated with stabilizers to prevent agglomeration.

The focus in much of the literature has been on ENM-induced cytotoxicity while in the present study, we observe minimal cytotoxicity after 1 h and 24 h exposure to ENMs at various doses up to 100 μg/ml. This suggests that ENMs are not altering mast cell activation through an apoptotic or necrotic pathway. A recent study from our laboratory demonstrated that mast cells are not highly phagocytic and therefore are not readily internalizing ENMs, which could account for the difference in cytotoxic responses compared to other immune cell types^48^. Dendritic cells, neutrophils, and macrophages are all shown to readily phagocytize particles, altering cell viability and function^52^. The current study demonstrates minimal Ag (20 and 110 nm) association and uptake by mast cells. Iron oxide (Fe_2_O_3_) had the highest level of cell association, consistent with previous finding from Toki *et al*.^53^. Internalization of selected ENMs by mast cells is a complex process that is likely dependent on a combination of physicochemical properties but may not be a contributing factor mediating mast cell degranulation.

Mast cell exposure to different ENMs resulted in varying degranulation responses, illustrating the importance of physicochemical properties. The literature supports our findings that a subset of ENMs are activating and others are inhibiting mast cell degranulation, which we infer are likely mediated through surface receptor-driven mechanisms. Identifying ENM properties that are functioning as immunomodulators, specifically in the activation of mast cells could provide knowledge for diseases associated with idiopathic mast cell activation disorders, in which the triggers are unknown but the responses are similar to what we observed following ENM exposures^54^. Future research needs to be performed to elucidate the receptors activated in response to ENM exposures, and the resulting signal transduction pathways that are likely modulating mast cell degranulation and potentially contributing to allergic immune responses.

## Methods

### Engineered nanoparticles (ENMs)

Silver nanoparticles sizes 5, 20, 50 and 110 nm (Ag-5, Ag-20, Ag-50, Ag-110) suspended in citrate buffer were purchased from NanoComposix at a concentration of 1 mg/ml. All other ENMs used in the study: titanium dioxide (TiO_2_), iron oxide (Fe_2_O_3_), magnesium oxide (MgO), boron nitrite sizes ultrafine (BN-UF) and microfine (BN-MF), zinc oxide (ZnO), silica dioxide sizes 30 nm (SiO_2_-30) and 60 nm (SiO_2_-60), and copper oxide (CuO) were purchased from US Research Nanomaterials, Inc and stored as a dried powder at 4 °C until ready for assay preparation. Two-dimensional boron nitride (BN) was synthesized by chemical exfoliation of bulk BN (purchased from Graphene supermarket) using a Branson ultrasonic tip (1/8″ diameter, 250 W)^55^,^56^. ZnO nanoparticles were synthesized using a polyol method through drop-wise addition of aqueous Zn acetate and poly-vinylpyrrolidone (PVP) at 100 °C^57^. Biocorona was formed by incubating Ag-20 (20 nm) with bovine serum albumin (BSA) at a concentration of 100 μg/ml for 1 hour at 37 °C. Following incubation, biocorona was pelleted by centrifugation for 10 min at a speed of 14,000 rpm and then re-suspending in 1 mg/ml of diH_2_O as previously described^11^.

### ENM characterization

ENMs diluted to 1 mg/ml in deionized (diH_2_O) water were sonicated for 30-second pulses at 35,000 joules (sonicator, Fisher Scientific). The hydrodynamic size and zeta potentials (ZetaSizer Nano, Malvern) in addition to the pH (Metler Toledo, Columbus, OH) of all ENMs were characterized in diH_2_O or HEPES biological buffer used in the experimental assays (N-2-hydroxyethylpiperazine-N-2-ethane sulfonic acid, 10 mM, pH 7.4). All measurements were performed with 3 individual samples at concentrations of 10 μg/ml. The primary size and shape were confirmed via transmission electron microscopy (TEM, Hitach H7600). Image J software was used to analyze size distribution. 100 particles per sample were counted by randomly surveying the entire TEM grid from multiple high magnification images. Image J was used to determine both area and Feret diameter (the greatest distance between two points on an objects’ boundary). The total particle number (N/g) and apparent surface area (m^2^/g) were calculated using the following equations:


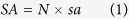



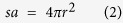



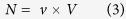



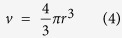



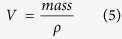


Equation (1) is used to calculate the total surface area by multiplying total number of particles by apparent surface area (*sa*) of each individual particle. Equation (2) is used to calculate surface area (*sa*) of each individual particle by multiplying 4 × π × radius^2^. Equation (3) is used to calculate total number of particles (N) by multiplying volume of a sphere for each individual particle (*v*) by the total volume of all particles (V, cm^3^). Equation (4) is used to calculate volume of a sphere for each individual particle (*v*) by multiplying 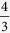 × π × radius^3^. Equation (5) is used to calculate the total volume of all particles (V, cm^3^) by dividing mass (g) by density of ENMs (ρ, g/cm^3^).

### Cell culture

Bone marrow-derived mast cells (BMMCs) were cultured from femoral stem cells isolated from C57BL/6 J mice (Jackson Laboratories, Bar Harbor, ME). Bone marrow stem cells pooled from 2 mice (4 femurs) were cultured at 37 °C and 5% CO_2_ for 4–6 weeks in the presence of IL-3. Mast cell population and maturity prior to experiments were confirmed by the expression on FcεRI and cKIT via flow cytometry (data not shown). Cells were cultured in RPMI media containing 10% FBS, 100 μg/ml streptomycin, 100 U/ml penicillin, 100 μg/ml primocin (Invivogen, San Diego, CA), 25 mM HEPES, 1.0 mM sodium pyruvate, nonessential amino acids (BioSource International, Camarillo, CA), 0.00035% 2-mercapto-ethanol, and 300 ng/ml purified recombinant mouse IL-3 (PeproTech, Rocky Hill, NJ). All animal procedures were approved and conducted in accordance with the National Institutes of Health guidelines and approved by the University of Colorado Denver Institutional Animal Care and Use Committee. All animals were treated humanely and with regard for alleviation of suffering.

### Cytotoxicity: Annexin V/ propidium iodide (PI) staining

Cytotoxicity of BMMCs following ENM exposure were evaluated by measuring the expression of early phase apoptotic marker Annexin V & late-phase marker propidium iodide (PI) via flow cytometry following manufacturer’s protocols (antibodies, BD Biosciences, San Jose, CA). BMMCs seeded at 5 × 10^4^ cells per well were treated with ENMs at concentrations 25 and 50 μg/ml for 1 h and 24 h and evaluated for cytotoxicity. The concentrations were decided based on the evaluation of other ENMs at similar concentrations in the literature as well as performed by the NIEHS Nano GO Consortium^58^. The values for Annexin V/PI represent percent apoptotic cells positive for the expression of Annexin V and PI (Annexin V^+^/PI^+^). Treatment with capsaicin at 100 μM was used as a positive control for early-phase apoptosis (Annexin V) and hydrogen peroxide (H_2_O_2_) at 10 mM was used as a positive control for late-phase apoptosis (PI). All experiments were performed in triplicate from 3 individual batches of mature mast cells grown in supplemented media (n = 3/group). Each batch of cells contains femoral bone marrow pooled from 2 mice.

### Mast cell degranulation assay

BMMC’s cultured for 4–6 weeks and seeded at 5 × 10^4^ cells per well in HEPES buffer were analyzed for β-hexosaminidase enzyme release following exposure of either ENMs or dinitrophenyl (DNP), as previously described^40^,^59^. Briefly, BMMC’s were sensitized overnight with anti-DNP IgE (Sigma- Aldrich, St. Louis MI) and treated with dinitrophenylated human serum albumin (DNP-HSA) (Sigma-Aldrich) for 30 min (100 ng/ml) to generate a positive control. Separately, non-sensitized BMMCs were exposed to ENMs at 25, 50, or 100 μg/ml concentrations (diluted to concentrations in diH_2_O) with or without the addition of a bovine serum albumin (BSA) biocorona for 1 h. Following incubation at 37 °C, supernatant and lysed cells were treated with *p*-nitrophenyl-N-acetyl-β-d-glucopyranoside, a substrate for N-acetyl-β-D-hexosaminidase (β-hexosaminidase), for 90 min and analyzed at 405 nm using an uv/vis spectrometer (BioTeck Instruments Inc., Winooski, VT). β-hexosaminidase release was calculated as percent total cell content after subtracting background release from untreated control groups and ENM-only control groups to ensure there was no interference with the biochemical assay. All experiments were performed in triplicate from 3 individual batches of mature mast cells grown in IL-3 supplemented media (n = 3/group).

### Osteopontin measurement

BMMCs seeded at 2 × 10^5^ cells per well were exposed to ENMs at 50 μg/ml for 24 h. Osteopontin (OPN) levels were measured in cell supernatant using a DuoSet ELISA kit (R&D Systems, Minneapolis, MN) following manufacturer’s protocol. Values are reported as an average of three technical replicates at pg/ml. All experiments were performed in triplicate from 3 individual batches of mature mast cells grown in supplemented media (n = 3/group). Each batch of cells contains femoral bone marrow pooled from 2 mice.

### Inductively coupled plasma-mass spectrometry (ICP-MS)

BMMCs seeded at 2 × 10^5^ were exposed to ENMs at 50 μg/ml for 1 h. Following exposure, samples were collected and centrifuged for 5 min at 1200 rpm, washed two times with PBS to remove excess ENMs, and dissolved in 6 ml of 2% HNO_3_. Metal concentrations from each ENM treatment were determined by inductively coupled plasma-mass spectrometry (ICP-MS, X series II, Thermo Scientific) using an internal standard containing Li, Y, and In with a resolution of 0.1 ppb. All experiments were performed in triplicate from 3 individual batches of mature mast cells grown in supplemented media (n = 3/group). Each batch of cells contains femoral bone marrow pooled from 2 mice.

### Statistical analysis

All graphs and analyses were performed using GraphPad Prism 5 software (GraphPad, San Diego, CA). Data in figures are represented as mean ± standard error of the mean (SEM). Statistical comparisons were performed by a one-way analysis of variance (ANOVA), with differences between groups assessed using a Bonferroni *post hoc* test. Correlation studies were performed using Spearman’s rank-order correlation test (non-parametric). Differences were considered statistically significant at *p* ≤ 0.05.

## Additional Information

**How to cite this article:** Johnson, M. M. *et al*. Contribution of engineered nanomaterials physicochemical properties to mast cell degranulation. *Sci. Rep.* **7**, 43570; doi: 10.1038/srep43570 (2017).

**Publisher's note:** Springer Nature remains neutral with regard to jurisdictional claims in published maps and institutional affiliations.

## Supplementary Material

Supplementary Information

## Figures and Tables

**Table 1:** Characterization of engineered nanomaterials (ENMs).

ENM	Primary Size (nm)	Zeta potential (mV)	pH	Hydrodynamic size (d.nm)		Surface area (m^2^/g)		Particle number (1 × 10^20^/g)	
				H_2_O	HEPES	H_2_O	HEPES	H_2_O	HEPES
SiO_2_-30	22 ± 6.6	−17	6.9	530 ± 68	299 ± 85	3.78	6.34	0.98	2.78
SiO_2_-60	64 ± 7.5	−19	6.8	816 ± 275	282 ± 74	2.10	3.80	0.01	0.60
TiO_2_	49 ± 7.2	21	6.7	696 ± 75	979 ± 380	1.34	1.15	0.10	0.06
CuO	92 ± 22.7	−12	7.0	73 ± 32	202 ± 24	11.0	2.69	1.69	1.70
BN-UF	50 ± 11.3	−17	7.4	400 ± 78	717 ± 266	4.39	3.20	1.05	0.31
BN-MF	117 ± 35.6	−19	7.0	373 ± 51	566 ± 135	3.50	3.34	0.50	0.36
MgO	95 ± 10.7	12	10.8	793 ± 180	566 ± 14	1.20	1.52	0.09	0.10
Fe_2_O_3_	28 ± 8.6	−8	6.7	251 ± 104	356 ± 82	2.91	5.45	0.06	0.57
ZnO	25 ± 6.1	−6	8.1	294 ± 37	621 ± 116	3.77	0.87	1.05	0.04
Ag-5	5.2 ± 0.9	−29	7.5	6.8 ± 0.5	324 ± 017	27.93	0.44	7260.00	0.53
Ag-20	20 ± 4.2	−38	7.8	16 ± 1	223 ± 29	18.50	1.51	1470.00	0.78
Ag-50	51.6 ± 6	−33	7.1	78 ± 23	300 ± 60	7.65	0.48	1600.00	0.67
Ag-110	110 ± 7.7	−36	7.2	107 ± 15	468 ± 209	4.69	1.10	25.50	0.33

Primary size (nm) determined by TEM and hydrodynamic size (d.nm) and zeta potential (mV) were determined by ZetaSizer Nano. Hydrodynamic surface area (m^2^/g) and particle number (1 × 10^20^/g) of ENMs were calculated using hydrodynamic size measured in de-ionized H_2_O and HEPES buffer. Values represent mean ± SEM (n = 3/group).

**Table 2:** Characterization of Ag-20 with the addition of a bovine serum albumin (BSA) biocorona.

Ag NPs +/− serum	Hydrodynamic size (d.nm)	Zeta potential (mV)
20 nm	20 ± 4.2	−38
20 nm + BSA	121.2 ± 12.8	−18

Hydrodynamic size (d.nm) and zeta potential (mV) were determined by ZetaSizer Nano. Values represent mean ± SEM (n = 3/group).
