# Supplementary material for: Contribution of engineered nanomaterials physicochemical properties to mast cell degranulation
Source: Sci Rep. 2017 Mar 6;7:43570. doi: 10.1038/srep43570 (PMC5337938; doi:10.1038/srep43570)
Supplement: Supplementary Information [file srep43570-s1.doc]

**Contribution of engineered nanomaterials physicochemical properties to mast cell degranulation**

Monica M. Johnson^1^, Ryan Mendoza^1^, Achyut J. Raghavendra^2,3^, Ramakrishna Podila^2,3^, Jared M. Brown^1^


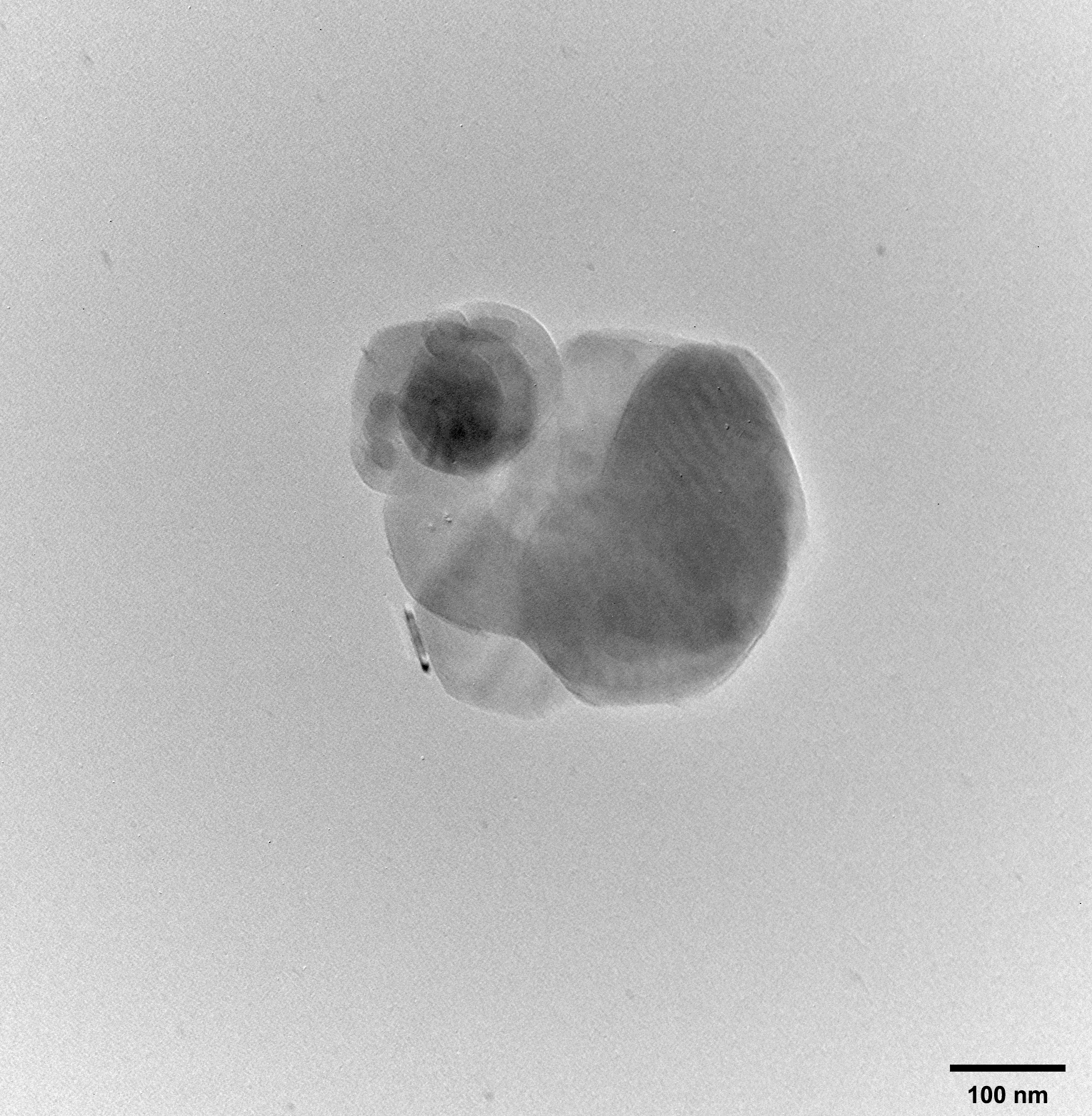


BN-MF


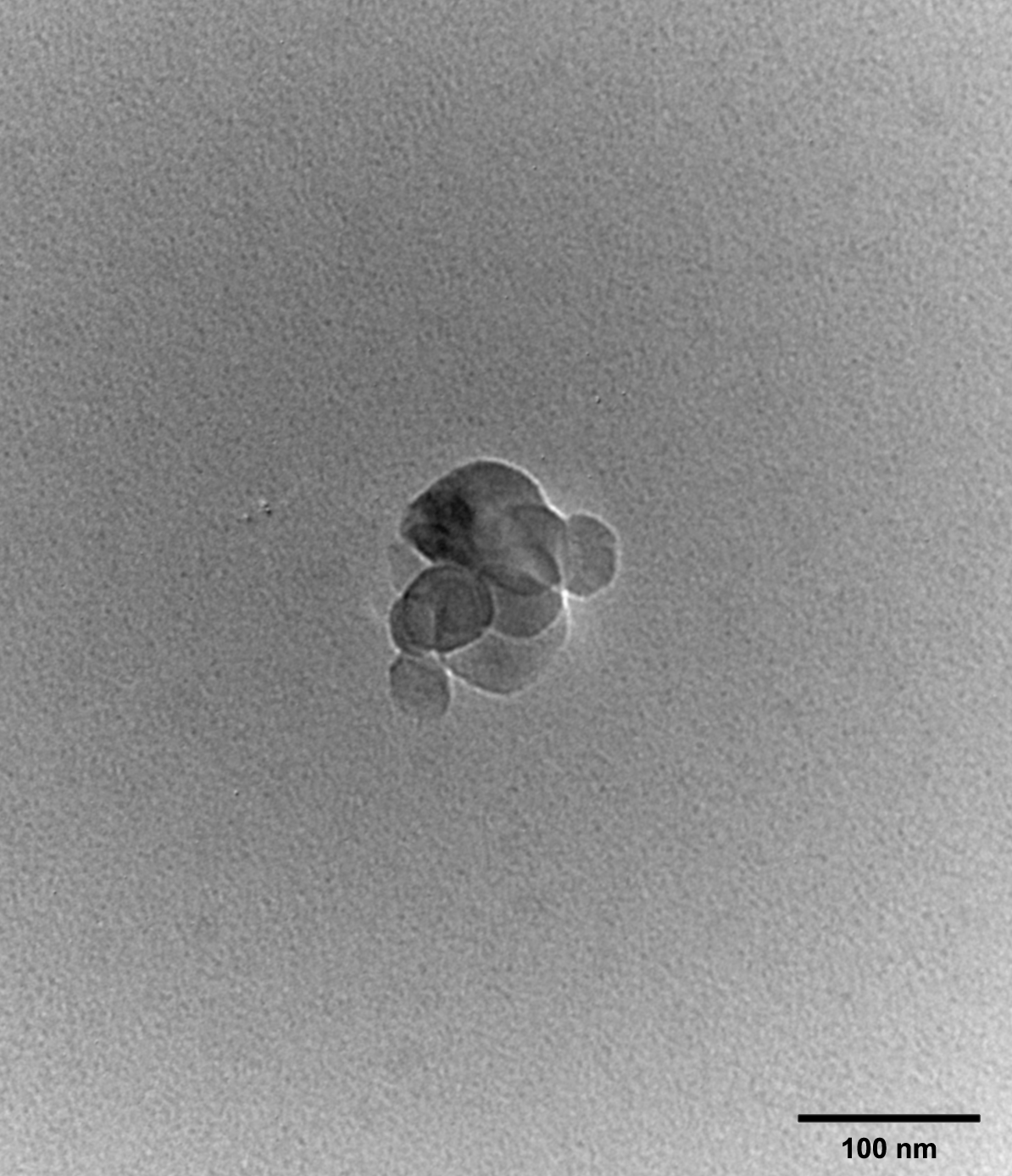


BN-UF


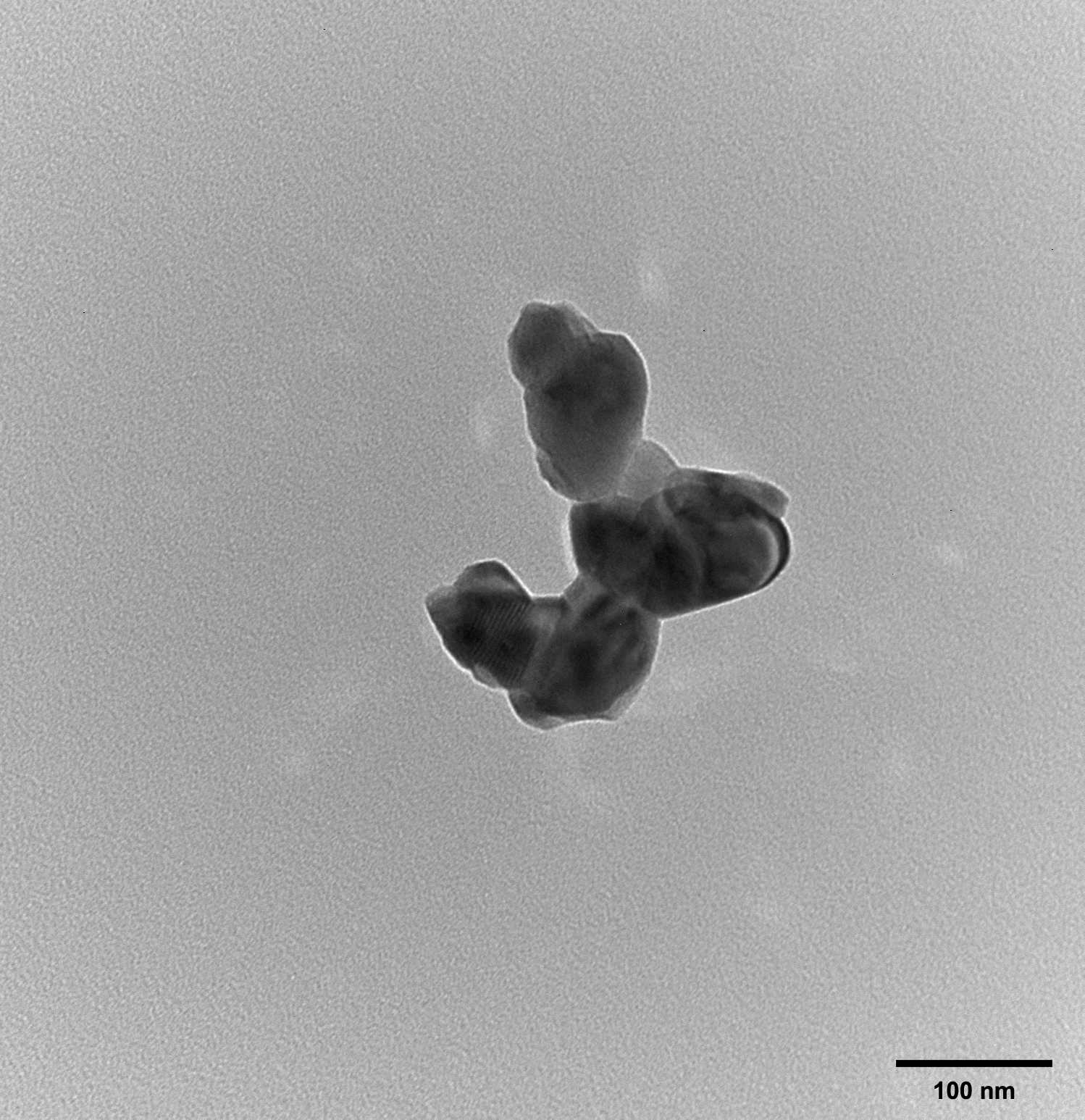


CuO


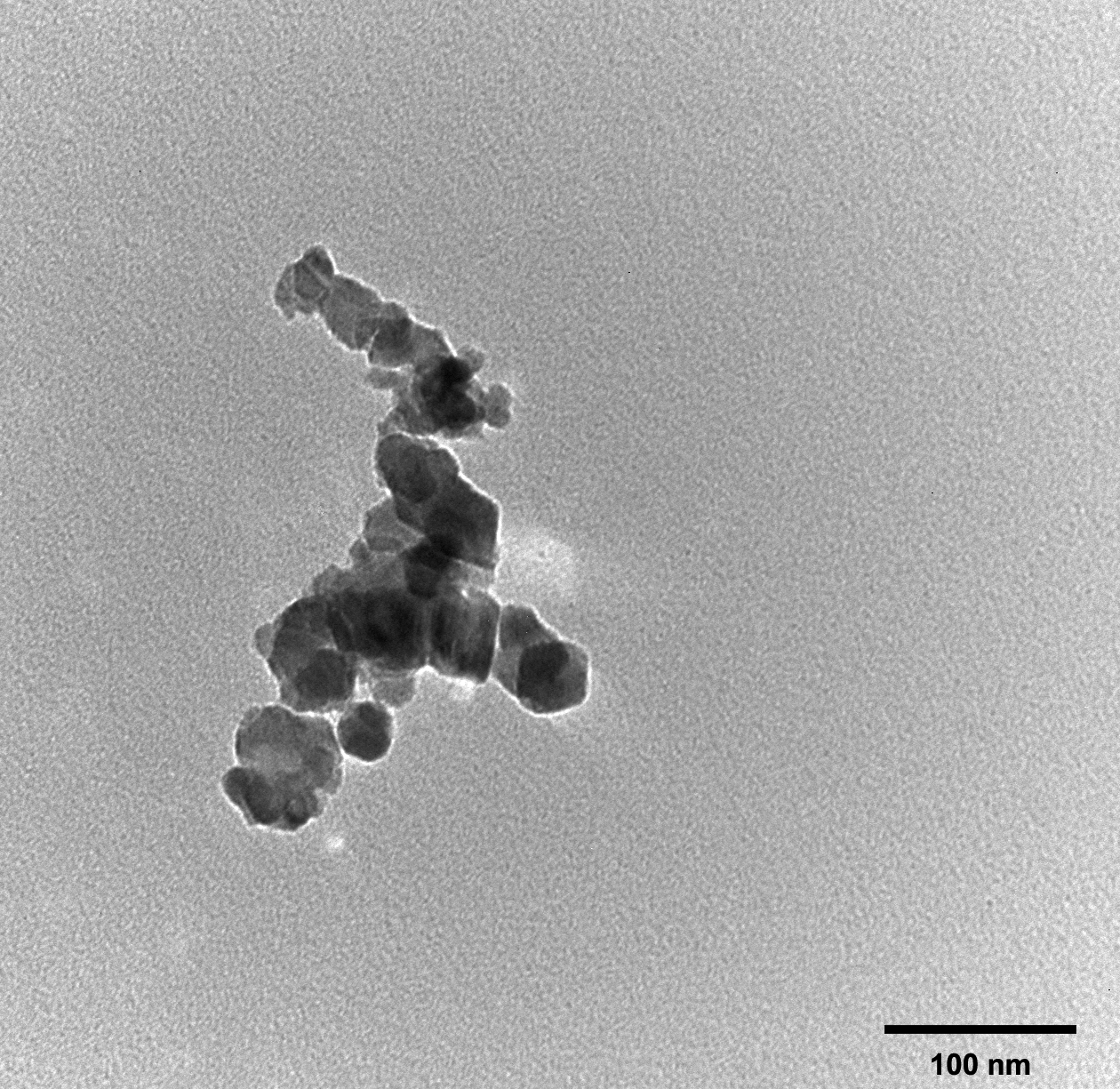


Fe_2_O_3_


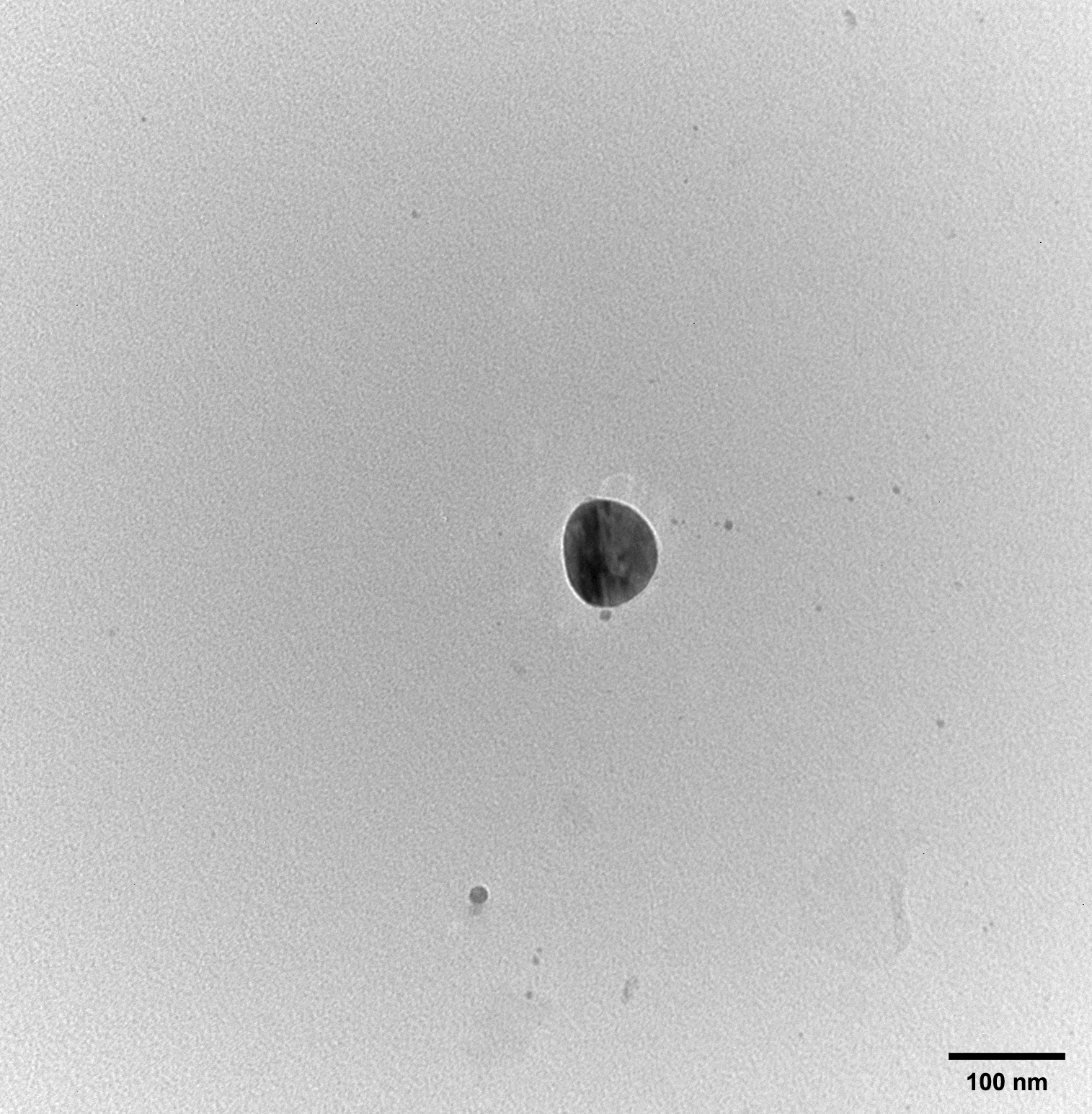


MgO


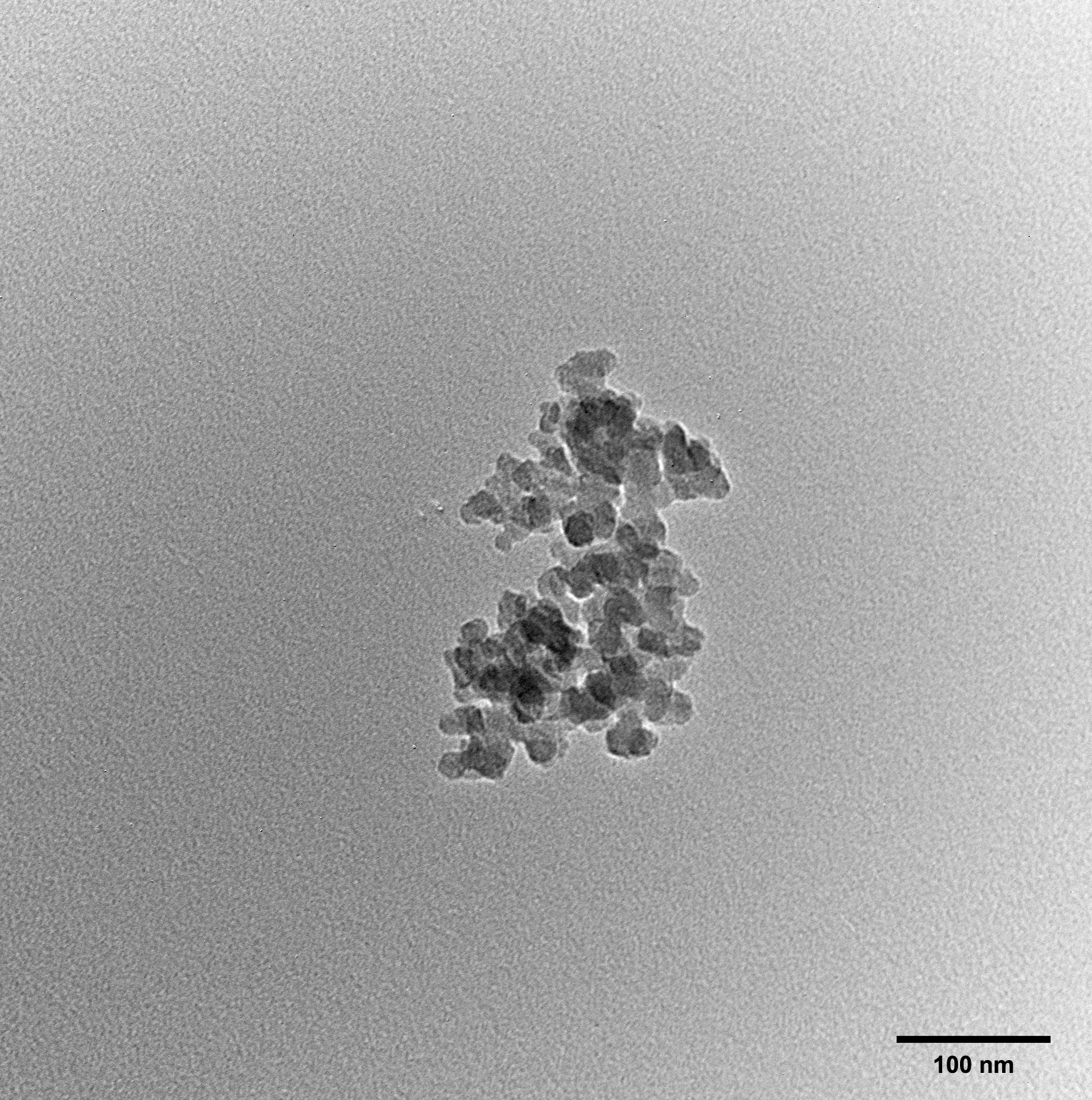


SiO_2_-30


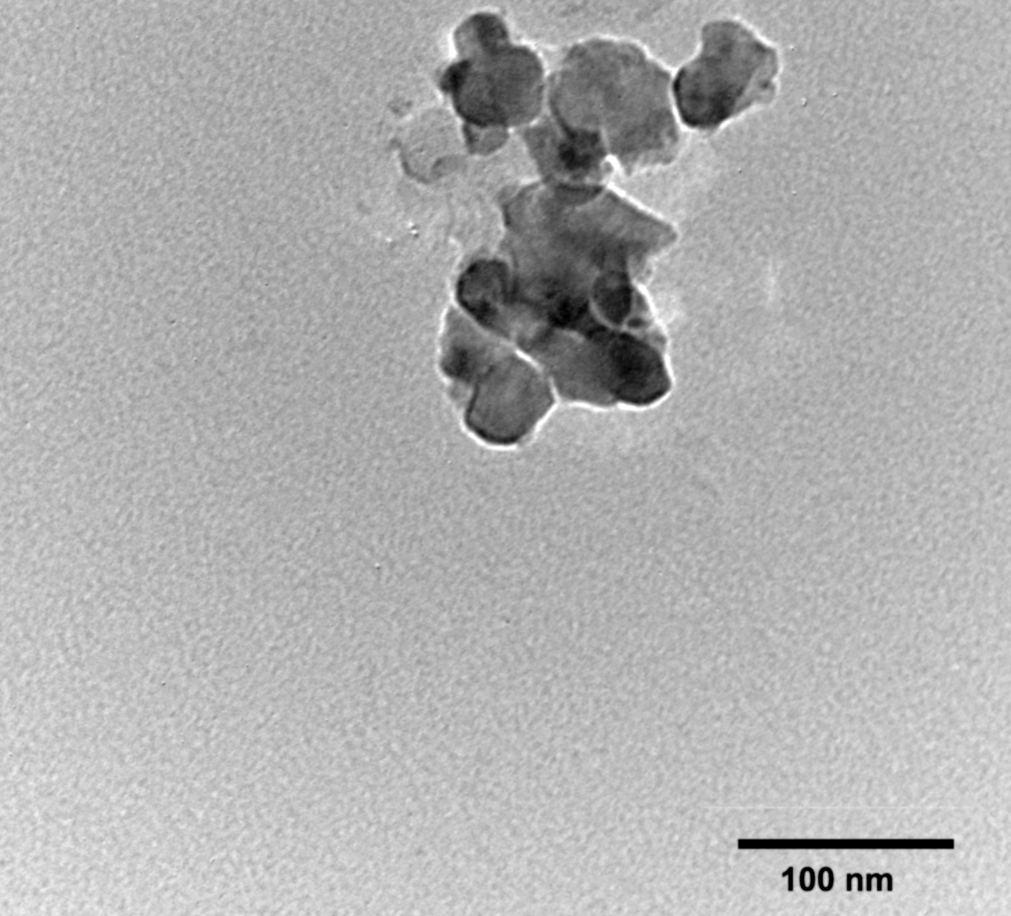


SiO_2_-60


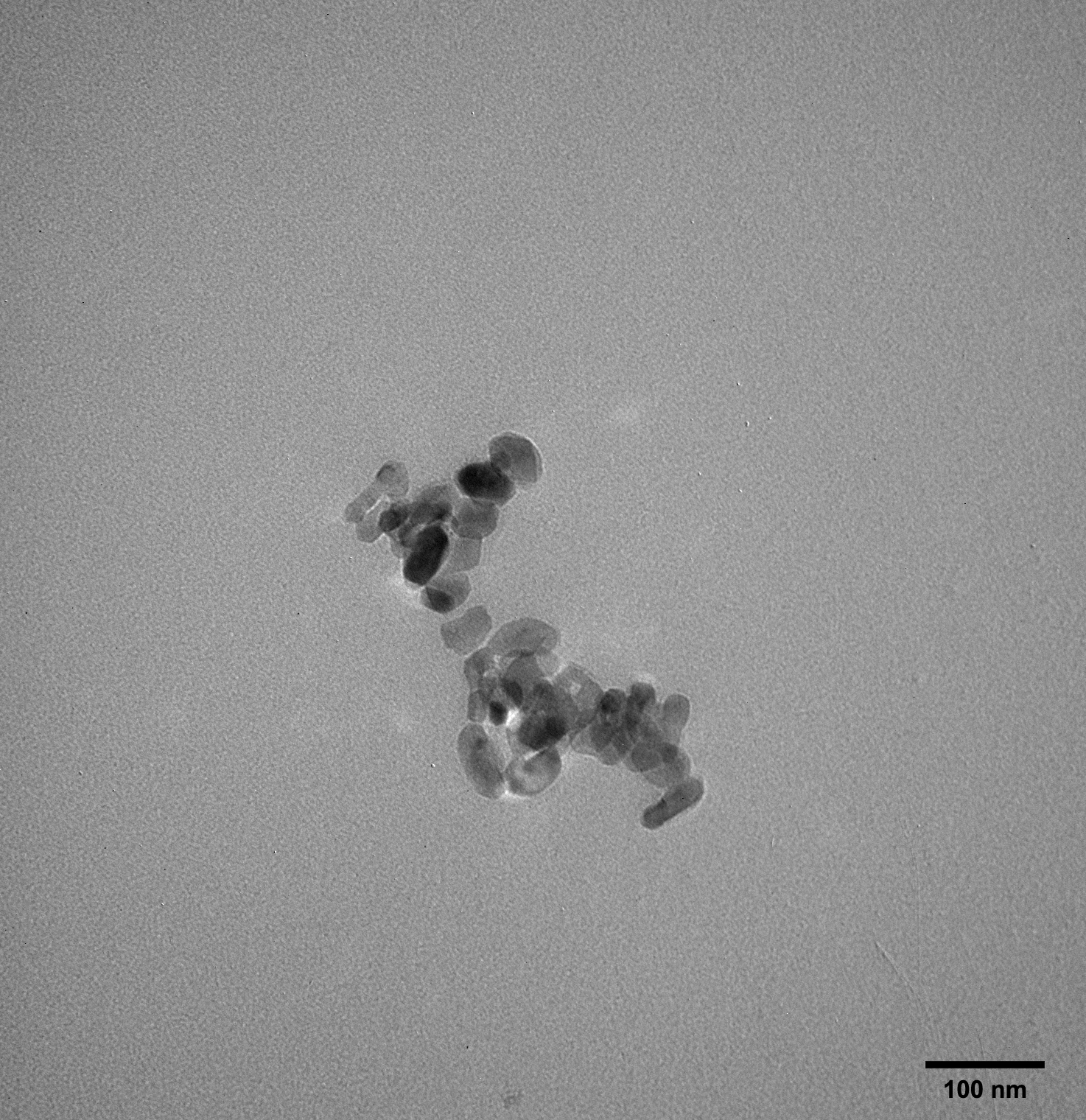


TiO_2_

ZnO


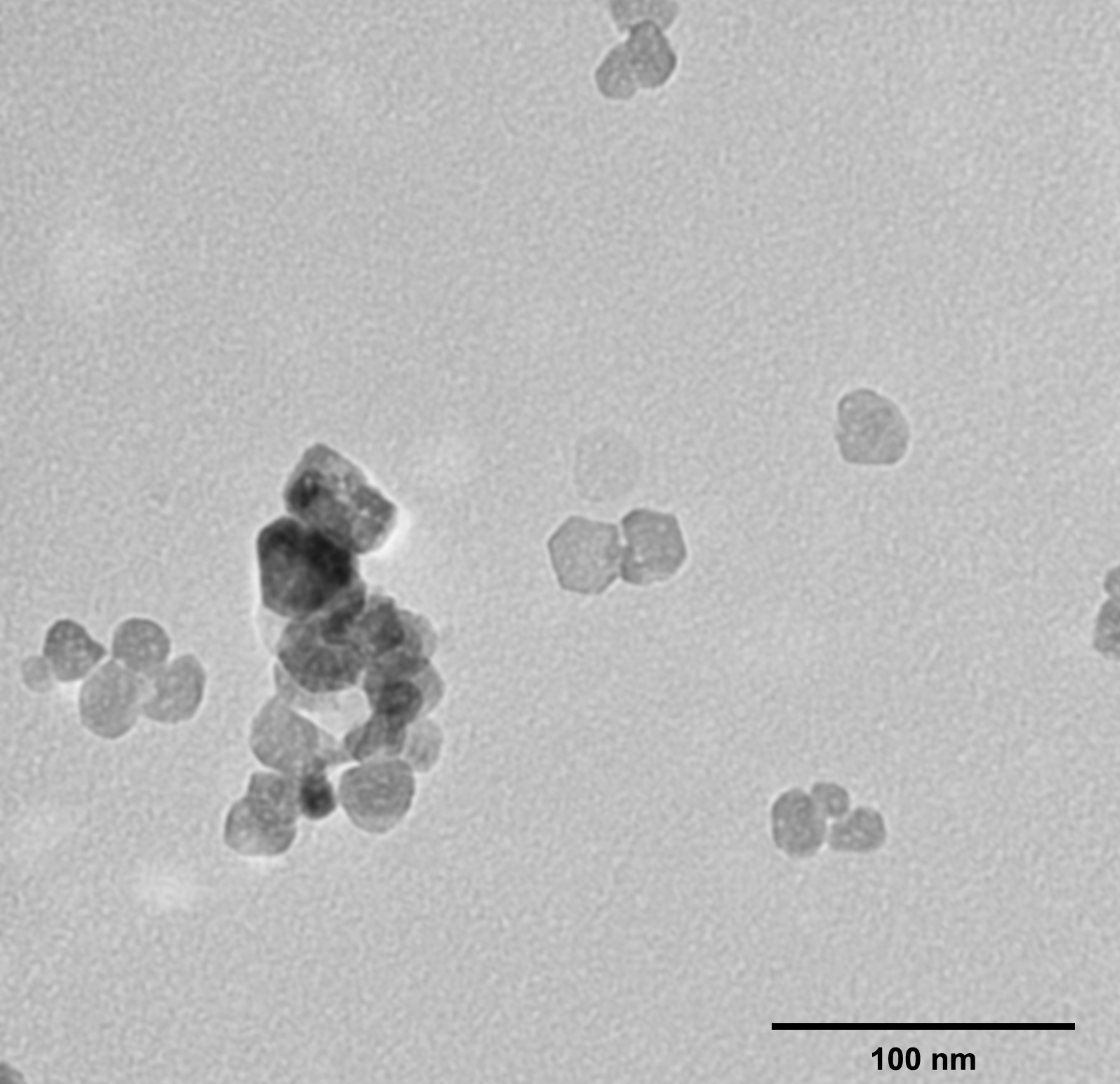


Ag-20

Ag-110


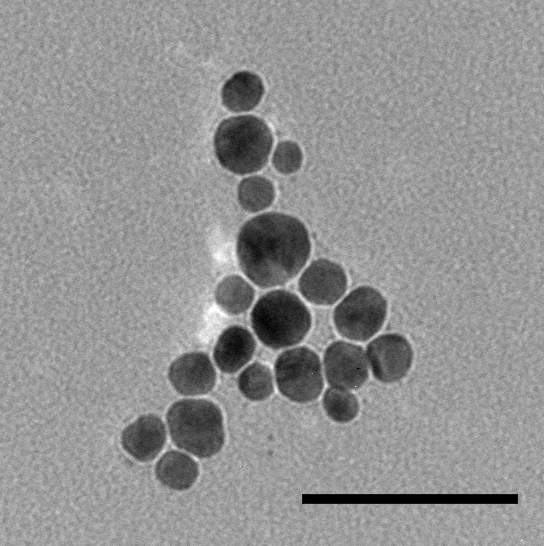


**100 nm**

**100 nm**


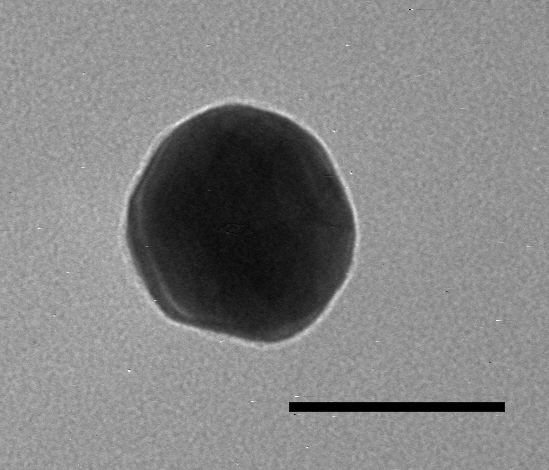


**100 nm**

**Supplemental Figure 1.** Representative TEM images demonstrating shape and size of ENMs. ENMs with various chemical compositions were evaluated including silica dioxide (SiO_2_-30 and SiO_2_-60), titanium dioxide (TiO_2_), copper oxide (CuO), zinc oxide (ZnO), magnesium oxide (MgO), boron nitride ultrafine (BN-UF) and microfine (BN-MF) sizes, iron oxide (Fe_2_O_3_), and silver nanoparticles (Ag-20 and Ag-110).

**Supplemental Figure 2**. Dose-dependent increase in ENM-mediated mast cell degranulation. Bone marrow-derived mast cells (BMMCs) were exposed to ENMs at a mass concentration of 25, 50, and 100 μg/ml for 1 h and evaluated for the release of b-hexosaminidase into the supernatant. Values are expressed as mean ± SEM normalized to non-treated control group (n=3/group).
